# Identification of Galectin-1 as a Critical Factor in Function of Mouse Mesenchymal Stromal Cell-Mediated Tumor Promotion

**DOI:** 10.1371/journal.pone.0041372

**Published:** 2012-07-23

**Authors:** Gábor János Szebeni, Éva Kriston-Pál, Péter Blazsó, Róbert László Katona, Julianna Novák, Enikő Szabó, Ágnes Czibula, Roberta Fajka-Boja, Beáta Hegyi, Ferenc Uher, László Krenács, Gabriella Joó, Éva Monostori

**Affiliations:** 1 Institute of Genetics, Biological Research Center, Hungarian Academy of Sciences, Szeged, Hungary; 2 Faculty of Health Sciences and Social Studies, University of Szeged, Szeged, Hungary; 3 Stem Cell Biology, National Blood Service, Budapest, Hungary; 4 Tumor Pathology and Molecular Diagnostics Laboratory, Szeged, Hungary; Cincinnati Children's Hospital Medical Center, United States of America

## Abstract

Bone marrow derived mesenchymal stromal cells (MSCs) have recently been implicated as one source of the tumor-associated stroma, which plays essential role in regulating tumor progression. In spite of the intensive research, the individual factors in MSCs controlling tumor progression have not been adequately defined. In the present study we have examined the role of galectin-1 (Gal-1), a protein highly expressed in tumors with poor prognosis, in MSCs in the course of tumor development. Co-transplantation of wild type MSCs with 4T1 mouse breast carcinoma cells enhances the incidence of palpable tumors, growth, vascularization and metastasis. It also reduces survival compared to animals treated with tumor cells alone or in combination with Gal-1 knockout MSCs. *In vitro* studies show that the absence of Gal-1 in MSCs does not affect the number of migrating MSCs toward the tumor cells, which is supported by the *in vivo* migration of intravenously injected MSCs into the tumor. Moreover, differentiation of endothelial cells into blood vessel-like structures strongly depends on the expression of Gal-1 in MSCs. Vital role of Gal-1 in MSCs has been further verified in Gal-1 knockout mice. By administering B16F10 melanoma cells into Gal-1 deficient animals, tumor growth is highly reduced compared to wild type animals. Nevertheless, co-injection of wild type but not Gal-1 deficient MSCs results in dramatic tumor growth and development.

These results confirm that galectin-1 is one of the critical factors in MSCs regulating tumor progression.

## Introduction

In spite of the increasing significance [1], [2], the origin of the tumor-associated non-tumor-cell elements (tumor-associated fibroblasts and endothelium) has not been determined decisively. As it has recently been shown, one source of the tumor-associated stroma (TAS) is bone marrow-derived mesenchymal stromal cells, which migrate into the solid tumor and there contribute to the establishment of TAS [3]. Also MSCs have been implicated in formation of tumor blood vessel endothelium partially by secreting angiogenic factors such as vascular endothelial growth factor (VEGF), fibroblast growth factor (FGF), platelet-derived growth factor (PDGF), and stromal-derived factor-1 (SDF-1) [4]. Moreover, they are able to differentiate into endothelial cells as well [5].

Exogenously administered MSCs migrate and specifically localize into tumors [6]–[8]. However, the effect of transplanted MSCs in term of tumor progression is still actively debated, since both tumor-promoting and tumor-moderating functions have been indicated [9]. Tumor promotion by MSCs has been primarily attributed to their immunosuppressive function [10] and neo-vascularization promoting effect [11]. All effects of MSCs in solid tumors can be explained by two mechanisms: 1) differentiation of the multipotent MSCs into tumor-associated tissue elements such as fibroblasts [12], tumor-associated blood vessel endothelium [5] and/or smooth muscle [13] or 2) MSCs are not stably associated with these sites, rather they affect tumorigenesis *via* producing various angiogenic (VEGF, PDGF, FGF) [4], immunosuppressive (TGF-β1, IDO, IL-10, PGE2) [14] and metastatic (CCL5) [15], [16] factors. These possibilities are not exclusive; however providing definite answer is difficult due to the lack of MSC specific molecular markers.

Galectin-1 (Gal-1) is an immunosuppressive and pro-angiogenic member of the β-galactoside-binding lectin family, galectins. Immunosuppressive function of Gal-1 has been confirmed in a number of *in vivo* and *in vitro* studies [17]. Targeted inhibition of Gal-1 expression or function in tumor cells provokes immune response against the tumor and subsequent tumor rejection [18], [19]. Also, Gal-1 has recently been implicated in growth and metastasis of solid tumors [20]. Accordingly, high expression of Gal-1 in the tumor cells and/or in TAS indicates poor prognosis of the disease [21]. Crucial role of Gal-1 in tumor angiogenesis has also been confirmed [22], [23]. Additionally, genetically engineered carcinoma-associated fibroblasts expressing low level of Gal-1 failed to help tumor progression [24]. High level of Gal-1 expression has been detected in MSCs [25] contributing to the T-cell regulating role of MSC *in vitro* [26].

Here we demonstrate an important role of MSC-derived Gal-1 in regulation of tumor growth and metastasis since the absence of Gal-1 expression results in the loss of tumor promoting effect of MSCs. *In vitro* studies show that the absence of Gal-1 in MSCs does not affect the number of migrating MSCs toward the tumor cells which is supported by the *in vivo* migration of intravenously injected MSCs into the tumor. Also the *in vitro* differentiation of endothelial cells into blood vessel-like structures strongly depends on the expression of Gal-1 in MSCs indicating its important role in tumor neo-angiogenesis. Vital role of MSC-derived Gal-1 in tumorigenesis has been further verified in Gal-1 knockout mice. Administering B16F10 melanoma into Gal-1 deficient animals, tumor growth is highly reduced compared to wild type animals. Nevertheless, co-injection of wild type but not Gal-1 deficient MSCs results in dramatic tumor progression confirming the essential role of Gal-1 expression in MSCs.

## Materials and Methods

### Ethics Statement

All mouse studies were done in accordance with national and international law and regulations of animal experiments and were reviewed and approved by the Institutional Animal Care and Use Committee of Biological Research Center, Hungarian Academy of Sciences.

### Cells

Mouse breast cancer cell line, 4T1 (ATCC CRL-2539, Lot No: 3306022) [27] and mouse melanoma cells, B16F10 (ATCC CRL-6475, a kind gift of Dr. R. Kiss, Free University of Brussels, Belgium) [28] were maintained in RPMI complemented with 10% FCS, mouse endothelioma cells, H5V [29] (obtained from C Vizler, Biological Research Center, Hungary) and all types of mouse MSCs (established in our laboratories) were cultured in DMEM containing 10% FCS in a humidified incubator with 5% CO_2_ at 37°C. All cell culture media and FCS were purchased from Invitrogen (Carlsbad, USA, www.invitrogen.com).

Isolation of MSCs from bone marrow and adipose tissue was carried out according to Peister et al. [30] with some modifications as described previously [31], [32]. Characterization of the MSC cultures was performed by cytofluorimetry (FACSCalibur, Becton Dickinson) using CD34-FITC, R-Phycoerythrin-conjugated anti-mouse CD44, CD73, CD90, Sca-1 and biotinylated CD3ε, CD45R/B220, CD11b, Ly-6G, TER-119 (BD Pharmingen, Franklin Lakes, USA, www.bd.com) followed by Streptavidin-PE (Sigma-Aldrich, St. Louis, USA www.sigmaaldrich.com). Evaluation of the data occurred with CellQuest software (Becton Dickinson).

Extra- and intracellular Gal-1 in cells was detected by cytofluorimetry in non-permeabilized and permabilized cells using goat anti-mouse Gal-1 (R&D Systems) and donkey anti-goat Ig-NL493 conjugate (R&D Systems).

Osteogenic and adipogenic differentiation was induced by culturing confluent MSCs for 2 weeks in appropriate medium as described previously [31]. Photomicrographs were taken with Olympus CKX41 inverted light microscope and Olympus Camedia C-5060 camera (Tokyo, Japan, www.olympus-global.com).

The various MSC cultures were designated in the paper as follows: bone marrow and adipose tissue MSCs isolated from wild type animals = wtMSC, and wtA-MSCs, respectively MSC from Gal-1 knockout animals = MSC^Gal-1−/−^, and A-MSC ^Gal-1−/−^, MSCs transfected with Gal-1 silencing or scrambled (control) RNAi constructs = siMSC or scMSC, respectively.

### Silencing galectin-1 in MSCs

The method for silencing of galectin-1 gene has been previously described [33]. The following modifications have been introduced: human (hGalec120si) and mouse (mGalec340si) galectin-1 specific and scrambled (hGalec120sc and mGalec340sc) oligonucleotides were used to produce the silencing siRNA molecules (Table S1), which were designed to knock-down human (GenBank accession number BC020675.1) and mouse (GenBank accession number NM_008495.2) Gal-1.

The resulted four plasmid constructs were as follows: pSNhG120si, pSNmG340si for galectin-1 knock-down and pSNhG120sc and pSNmG340sc for control experiments, respectively. Mouse wtMSCs (10^6^ cells) were co-nucleofected with the combination of 1+1 µg pSNGh120si and pSNmG340si for silencing or identical amounts of pSNhG120sc and pSNmG340sc plasmids for obtaining controls. Nucleofection was performed on an Amaxa Nucleofector with the A-027 program and MEF Nucleofector Kit 1 (Lonza Amaxa, Walkersville, USA, www.lonzabio.com). Clone selection lasted 11 days in DMEM, 10% FBS, 5% Horse Serum, 1× NEAA, 1× PenStrep, 1× GlutaMAX (all medium components were obtained from Invitrogen, and 1 mg/ml G418 (Sigma-Aldrich, St. Luis, USA).

### Animal model

Number of mice included into each experimental group is indicated under the particular figure legend. The in vivo experiments were repeated independently three times and representative of the three experiments have been presented in the paper.

Female Balb/C and male C57BL/6 or Gal-1 knockout (strain: B6.Cg-*Lgals1* ^tm1Rob^/J, 006337, Jackson Laboratory, Bar Harbor, USA, www.jax.org) as well as X-SCID (strain: B6;129S7-*Il7r^tm1Imx^*/J, Jackson Laboratory, Bar Harbor, USA) mice (8–10 week old) were injected orthotopically with 4T1 breast carcinoma cells or subcutaneously with B16F10 melanoma cells into the right flank without or in combination with MSC^Gal-1−/−^ or MSCs expressing various amount of Gal-1 (wtMSC, scMSC or siMSC). In another model, Balb/C mice were orthotopically injected with 10^5^ 4T1 cells and when tumors became palpable, MSCs were administered intravenously as indicated. The animals were housed individually or with littermates of identical sex and had free access to food and water.

Tumors were evaluated macroscopically by the following parameters: 1) Tumor size was measured at least twice a week with a precision caliper and calculated according to the formula: *d^2^×D×0.5* [19], where *d* and *D* are the minor and major diameters, respectively; 2) incidence of palpable tumors was determined by the daily monitoring of animals in each experimental group; 3) after euthanizing the animals, weights of the excised primary tumors and lungs were measured after 4% formaldehyde fixation (Molar Chemicals, Budapest, Hungary, www.molar.hu); 4) metastatic nodules on the surface of lungs were counted under a stereo microscope (Leitz Wetzlar, Germany, www.ernst-leitz-wetzlar.de); 5) survival of experimental animals was estimated using Kaplan-Meier plot analysis.

### Characterization of Gal-1 expression by Western blotting and QPCR

#### SDS-PAGE and Western Blot analysis

Cell lysates (10^5^ MSCs, 2×10^5^ 4T1 or B16F10 cell equivalent/sample) were analyzed by Western blotting after running the samples on a 12%–17.5% gradient SDS-polyacrylamide gel (SDS-PAGE) then electro-transferred onto nitrocellulose membrane (Whatman® Protran, Kent, UK, www.whatman.com ®). After blocking with 3% gelatin (Sigma-Aldrich, St. Luis, USA) in TBS plus 0.05% Tween® 20 (Sigma-Aldrich, St. Luis, USA), the membrane was incubated with polyclonal rabbit anti-Gal-1 and HRP conjugated anti-rabbit immunoglobulin (Dako, Glostrup, Denmark, www.dako.com). Rabbit anti-Gal-1 was produced in our laboratory (for specificity control see Fig. S3). It reacted with monomer and dimer recombinant human Gal-1, gave one reactive band in wtMSC at the proper MW and none in MSC^Gal-1−/−^. Rabbit anti-β-actin (Abcam, Cambridge, UK, www.abcam.com) was used as loading control. Immunoreactive proteins were visualized using the ECL Plus detection system (GE Healthcare Amersham™ Waukesha, USA, www.gelifesciences.com) on Agfa X-ray film (Mortsel, Belgium www.agfa.com). Molecular mass standard proteins: Page Ruler™ Prestained Protein Ladder (Fermentas, Glen Burnie, USA, www.fermentas.com).

#### QPCR

Total RNA was extracted using RNeasy Plus RNA isolation kit (QIAGEN, Germantown, USA, www.qiagen.com) according to manufacturer's instruction. cDNA was obtained using GoScript™ Reverse Transcriptase (Promega Corporation, Madison, USA, www.promega.com) from 2 µg of template total RNA per reaction. QRT-PCR was performed in triplicates using TaqMan® Gene Expression Master Mix (Applied Biosystems Inc, Foster City, USA, www.appliedbiosystems.com) for Lgals1, Ptgs2, Tgfb1 and IL10 and AccuPower® 2× Greenstar qPCR Master Mix (Bioneer, Daedeok-gu, Daejeon, South Korea) for VEGFA, angiopoietin1, ORP150 and BEX2 in RotoGene3000 instrument (Corbett Life Science, Sydney, Australia). Relative quantification of gene expression was determined by comparison of threshold values. All results were normalized to GAPDH. TaqMan® Gene Expression Assays were commercially available for Lgals1 (Mm00839408_g1), Ptgs2 (Mm00478374_m1), Tgfb1 (Mm01178820_m1), IL10 (Mm00439614_m1, GAPDH (Mm99999915_g1), and QPCR primers were designed using Universal ProbeLibrary Assay Design program (Roche Applied Science, https://www.roche-applied-,science.com/sis/rtpcr/upl/index.jsp?id=UP030000) for VEGFA angipoietin1, ORP150 and BEX2. VEGFA fwd: aaaaacgaaagcgcaagaaa, rev: tttctccgctctgaacaagg; Angiopoietin fwd: cggatttctcttcccagaaac, rev: tccgacttcatattttccacaa; ORP150 fwd: tggcgtgctcagtttagaca, rev:agagtagattcttcctctgggcta; Bex2 fwd: actacgccgcaagggatag, rev: tttcacgccttgttccactt; GAPDH fwd: tttgatgttagtggggtctcg, rev: agcttgtcatcaacgggaag.

### Histology and Biometrics

Paraffin embedded tissue sections: Embedding, processing and Hematoxylin and Eosin (Sigma-Aldrich, St. Luis, USA) staining was carried out as standard protocols. Microvessel density (MVD) was analyzed with morphological means and with visual inspection of areas filled with red blood cells using a microscope (Carl Zeiss Axio Imager.Z1 and Carl Zeiss AxioCam MRc5 camera, Oberkochen, Germany, www.zeiss.com) and evaluated with AxioVision software (AxioVs40 V 4.6.3.0 Carl Zeiss Imaging Solutions GmbH) based on 10 randomly selected non-overlapping fields of 3 different samples (vascularized (vasc.) area (%) = average vasc. area (µm^2^)/field (µm^2^)×100). Relative microvessel density (MVD^rel^) was calculated by the following equation MVD^rel^ = MVD^(4T1+MSC)^/MVD^4T1^, where MVD^(4T1+MSC)^ = microvessel density of tumors initiated in the presence of 4T1 breast carcinoma and MSCs, MVD^4T1^ = microvessel density of tumors initiated with injection of 4T1 breast carcinoma alone.

Percentage of lung metastatic area was determined by the measurement of cumulated macrometastatic areas divided with the total lung tissue area ×100.

Frozen tissue sections (7 µm thick) were prepared according to the standard protocol. To track the localization of MSCs within the frozen tissue sections MSCs were pre-labeled with CM-DiI (Invitrogen, www.invitrogen.com). The sections were counter-stained with DAPI (Vector Laboratories, Burlingame, USA, www.vectorlabs.com) and mounted in Fluoromount G (Southern Biotech). CM-DiI labeled MSCs were visualized using laser scanning confocal microscope (Olympus FV 1000, Olympus Holding Europa GmbH, Hamburg, Germany, www.olympus-europa.com).

### *In vitro* capillary formation assay

MSCs were pre-labeled with CM-DiI according to the manufacturer's protocol. Then they were co-cultured with unlabeled mouse heart capillary endothelial cells, H5V in a ratio of 1∶1 in a 24-well plate (Orange Scientific, Braine-l'Alleud, Belgium) in the presence or absence of 30 mM sucrose (Sigma, St. Luis, USA) or thiodigalactoside (TDG, Carbosynth LTD). Separately cultured MSCs or H5V cells served as controls. Capillary formation was evaluated as follows: five randomly selected non-overlapping areas of co-cultured cells were examined by fluorescence microscope (Olympus IX81, Olympus Holding Europa GmbH, Hamburg, Germany, www.olympus-europa.com). Tubes were quantified by measuring the length of capillary-like structures with CellR Imaging Software Software (Olympus Holding Europa GmbH, Hamburg, Germany) after 3 days of cell cultures.

### *In vitro* migration assay of MSCs

2D migration test was performed in ibiTreat Ibidi µ-Dish ^35 mm,high^ Grid-500 culture (Ibidi, München, Germany, www.ibidi.com) dish. The two insert reservoirs were plated with 10^4^ MSCs pre-labeled with CM-DiI and 3×10^4^ 4T1 breast carcinoma cells in 70 µl of culture medium, respectively. Proliferation of the cells was blocked with 5 mM hydroxyurea (Sigma-Aldrich, St. Luis, USA) for 8 hours. At the starting time point the inserts were removed and migration was let for 16 hours in cell culture medium containing 5 mM hydroxyurea. The migration was stopped by fixing the cells with 4% paraformaldehyde (Sigma-Aldrich, St. Luis, USA) in PBS for 4 minutes at room temperature then washed with PBS. The nuclei of the cells were stained with Hoechst33342 fluorescent dye (Sigma-Aldrich, St. Luis, USA) for 30 min at 37°C. Snapshot photographs of initial and end state was taken with fluorescent microscope (Olympus IX81). Migrating cells were counted in 5 fields of 3 independent experiments and the migration distance was analyzed by ImageJ software (free access provided by NIH).

### Statistical analysis

Statistical analysis was performed using Student's t-test to evaluate the statistical significance (set at * p<0.05, ** p<0.01, *** p<0.001) between two given experimental groups.

## Results

### Characterization of MSCs and tumor cell lines

All types of MSCs including those of wild type (wtMSC), Gal-1 knocked down (siMSC), control cells transfected with scrambled RNA (scMSC) and Gal-1 knockout (MSC^Gal-1−/−^) cells expressed CD44, CD73, CD90 and Sca-1 (Fig. S1) but not markers of cells of hematopoietic origin, CD34, CD45R, Ly6G, CD3, CD11b, TER119 (data not shown), and differentiated into adipogenic and osteogenic directions (Fig. S2). Western blotting analysis showed that wt and scMSCs expressed abundant, siMSCs low level and MSC^Gal-1−/−^ none of Gal-1 (Fig. S3A). Tumor cell lines, 4T1 breast carcinoma and B16F10 melanoma cells also expressed Gal-1 although Gal-1 production in B16F10 was lower than in 4T1 (Fig. S3B and C). As Gal-1 level in MSCs and tumor cell lines is critical, it was quantified using QPCR. As shown on Fig. S3C 4T1 expressed 2 fold Gal-1 as wtMSC, while B16F10 melanoma cells produced only 20% of Gal-1 in wtMSC. Another crucial point is the localization of Gal-1 in MSCs as Gal-1 may function as intra- or extracellular factor. Fig. S3D shows that Gal-1 is present in both cellular compartments, however amount of secreted Gal-1 on B16F10 cell surface is less than on 4T1 (Fig. S3D).

MSCs produce tumor growth regulating factors other than Gal-1. Therefore the major factors affecting tumor growth, COX2 (PGE2 producing enzyme), TGF-β1, angiopoietin1, IL-10 and VEGF in wtMSCs and MSC^Gal-1−/−^ were analyzed. The expressions of these factors showed that COX2, TGF-β1 and angiopoietin1 mRNAs were expressed at similar level and IL-10 was not produced in either MSC lines. VEGF expression was slightly higher in MSC^Gal-1−/−^ than in wtMSCs (Fig. S3E). Recent papers [34], [35] implicated ORP150, a major pro-angiogenic gene and BEX2 (brain-expressed X-linked gene) in Gal-1 triggered pathways of *in vivo* and *in vitro* angiogenesis in glioblastoma tumors. Due to the importance of these gene products regulating tumor vascularization and being intimate signaling target for Gal-1 in brain tumors, QPCR experiments were carried out in wtMSCs and MSC^Gal-1−/−^ cells. As showed on Fig. S3F BEX2 was not expressed in the cells irrespective their Gal-1 expression. Although ORP150 was expressed, its expression did not depend on the presence or absence of Gal-1 in MSCs.

### Role of MSC-derived Gal-1 in MSC localization in the tumor tissue

**Figure 1:**
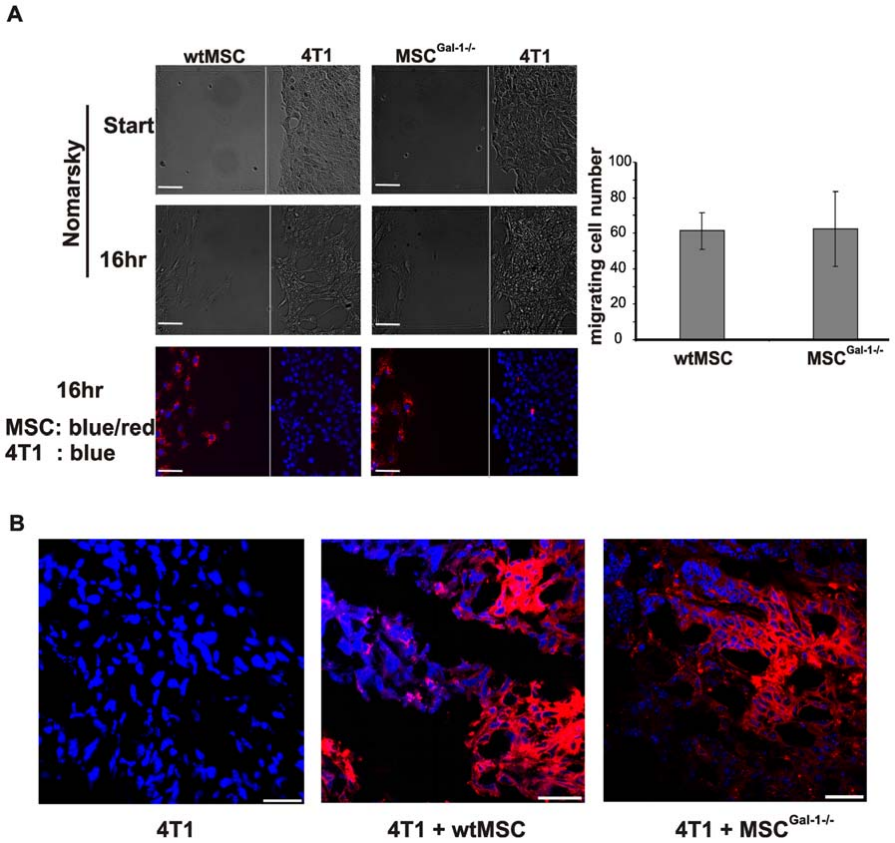
Galectin-1 expression in MSCs does not affect their localization within the tumor. (A) Migration of CM-DiI labelled wtMSCs and MSC^Gal-1−/−^ (red) toward 4T1 tumor cells i*n vitro* was followed for 16 h in Ibidi dish. All nuclei were visualized with Hoechst33342 (blue), and images were taken with fluorescent microscope. MSCs migrating to the cell free zone were counted and the average of 3 independent experiments was presented on the diagram (right panel). (B) Female Balb/C mice were challenged by 4T1 cells alone (10^3^ cells) (left) or in combination with 10^5^ wtMSC (middle) or MSC^Gal-1−/−^ (right) pre-labeled with CM/DiI (red). Cryosections of primary tumors were counter-stained with DAPI (blue) and analyzed with a confocal microscope. Representative images of 60 sections obtained from 3 independent experiments are shown. Scale bar: 30 µm.

To determine whether Gal-1 expression in MSCs contributed to the localization and survival of MSCs within the tumor environment, first an *in vitro* migration assay was carried out (Fig. 1A). Neither absence of Gal-1 in MSC^Gal-1−/−^ (Fig. 1A) nor reduction of Gal-1 in siMSCs (data not shown) affected the number of the migrating cells (Fig. 1A graph). There was no migration observed when MSCs were cultured alone in the migration plate (data not shown). Accordingly, *in vivo* migration of intravenously injected MSCs (expressing fluorescence protein, Venus) into pre-existing tumor bearing mice was not influenced by expression of Gal-1 when measured on the 7^th^ day following MSC injection (data not shown). Analysis of the frozen tumor tissue sections obtained from animals co-injected with 4T1 breast carcinoma cells and fluorescent dye labeled wtMSCs or MSC^Gal-1−/−^ showed no difference between the localization of the different MSCs (Fig. 1B). The siMSCs and scMSCs persisted in the tumor similarly to wtMSCs (data not shown).

### Gal-1-dependent enhancement of tumor growth

*In vitro* analysis of tumor cell growth in the presence of wt or MSC^Gal-1−/−^ was carried out. Neither supernatant of MSCs nor their direct interaction with tumor cells in co-culture affected growth of 4T1 or B16F10 tumor cells regardless of their Gal-1 expression (data not shown) indicating that MSCs did not directly act on tumor cell proliferation.

**Figure 2:**
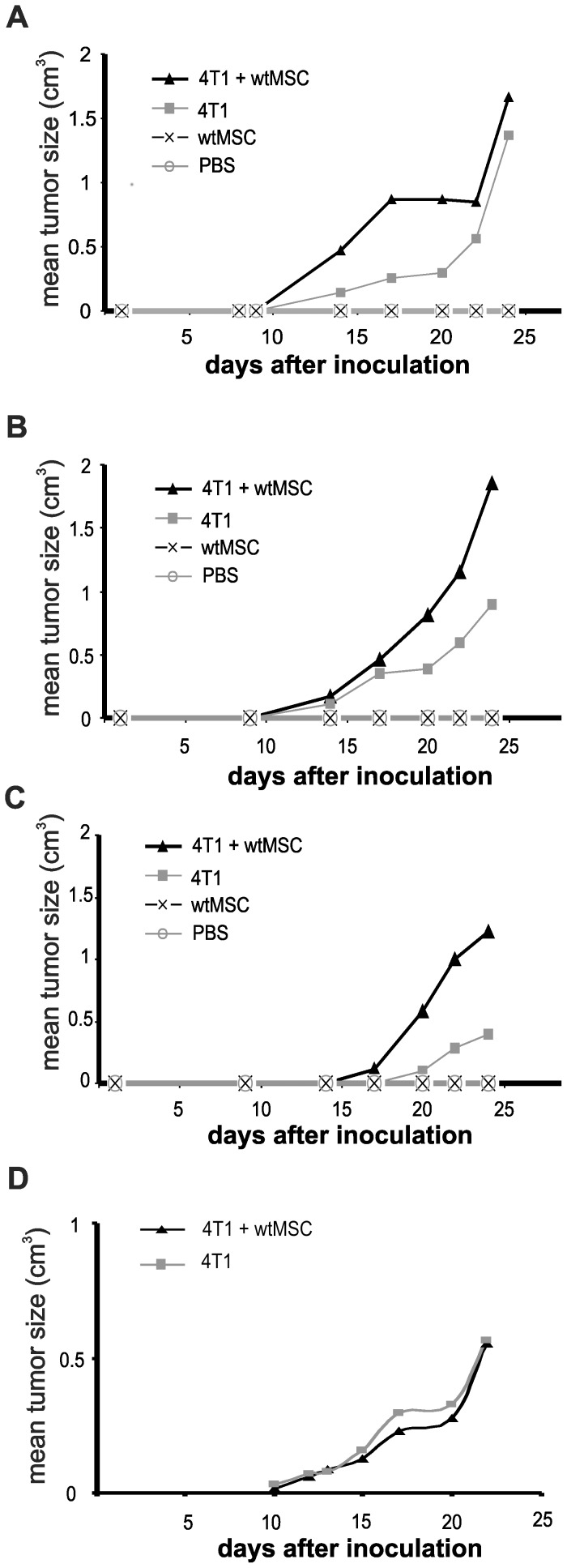
Tumor promoting activity of MSCs depends on the number of MSCs and ratio of MSCs/tumor cells injected. Different numbers of 4T1 breast carcinoma cells (A: 10^5^; B and D: 10^4^; C:10^3^) were injected with or without 10^5^ (A–C) or 10^4^ (D) MSCs into Balb/C female mice and tumor size was regularly measured with a special caliper. n = 5.

**Figure 3:**
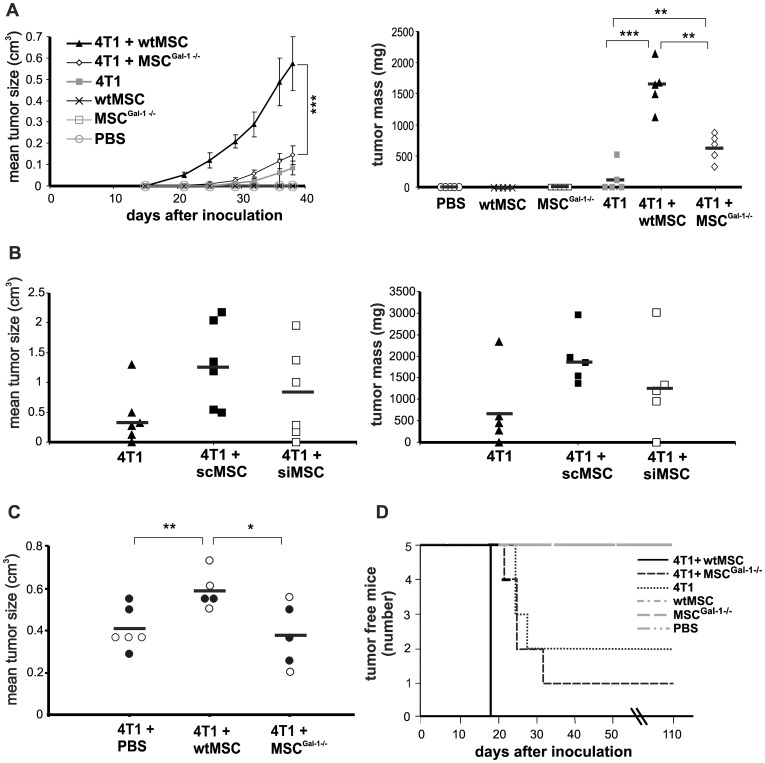
Deficiency of Gal-1 in knockout MSCs diminishes the tumor promoting effect of MSCs. Female Balb/C mice were challenged by orthotopic inoculation of 10^3^ 4T1 cells alone or in combination with 10^5^ wtMSCs or MSC^Gal-1−/−^ (A) or scMSCs or siMSCs (B). C) Tumor bearing (initiated by injection of 10^5^ 4T1 cells) mice were injected intravenously once with 10^6^ (open circles) or twice with 3×10^5^ (black circles) wtMSCs or MSCGal^-1−/−^. Tumor size (A left panel) was monitored daily or on the 40^th^ (B left panel) or 20^th^ day (C) after tumor initiation. Tumor mass was determined by measuring the formaldehyde fixed primary tumors isolated from sacrificed mice (A and B, right panels). Control mice were injected with PBS or MSCs without tumor cells. Data represent the mean ± SD. Significance on A left graph was calculated only for data on day 38. Number of animals in the experimental groups: A left panel: n = 5, A right panel: n = 5, B left panel: n = 6, B right panel: n = 5, C n = 5–6. ** p<0.01, *** p<0.001. D) Tumor incidence of mice challenged with 10^3^ 4T1 cells alone or in the combination with 10^5^ wtMSCs or MSC^Gal-1−/−^ was evaluated using Kaplan-Meier analysis. Control mice were injected with PBS, wtMSC or MSC^Gal-1−/−^. Mice were considered as tumor bearing when the tumor was palpable. Tumor free animals were surveyed up to 110 days. n = 5.

Balb/C mice were injected with syngeneic 4T1 breast carcinoma cells with or without wtMSCs or MSCs^Gal-1−/−^. MSC-tumor cell ratio varied as 1∶1, 10∶1 and 100∶1. MSC number was kept at 10^5^ and tumor cell number was changed. In all variations wt MSCs supported tumor growth. In contrast, when MSC number was decreased to 10^4^, in spite of the MSC-tumor cell ratio remained at 1∶1, MSC failed to affect tumor growth (Fig. 2). This result indicated that MSC number used to modulate tumor growth was a crucial point when playing role in early tumor development. Tumor cells and MSCs were applied in 1∶100 ratio in the further experiments, since it was the most effective combination in this setup. Tumor volume and weights were increased >5-fold (Fig. 3A left) and >7-fold (Fig. 3A right), respectively by wtMSCs on the 40^th^ day and the tumors were palpable much earlier (20^th^ *versus* 32^nd^ day) compared to that of induced by tumor cells alone. In contrast to wild type MSCs, co-injection of Gal-1 deficient MSC did not affect tumor development either in size, timing (Fig. 3A, left) or weight (Fig. 3A, right). Co-injection of siMSCs expressing low but detectable amount of Gal-1 (Fig. S3A) resulted in a reduction of tumor growth compared to the effect of control scMSCs (Fig. 3B). This result was reproducible but not significant indicating that low amount of Gal-1 in MSCs was sufficient to exert some tumor promoting effect. MSCs alone did not generate tumor development in 110 days follow up (Fig. 3A and Fig. 3D).

Whether MSC and MSC-derived Gal-1 affected growth of pre-existing tumors, mice carrying palpable breast tumor were transplanted with a single or a couple of injection with 10^6^ or 3×10^5^ MSCs, respectively. As Fig. 3C shows, injection of wtMSCs resulted in significantly higher tumor growth as compared to mice untreated with MSCs or treated with Gal-1 knockout MSCs indicating that Gal-1 in MSCs contribute to the MSCs' tumor-promoting activity in pre-existing tumors as well.

Analysis of incidence of palpable tumor showed that all mice, co-administered with 4T1 and wtMSC, developed tumors within 18 days (Fig. 3D). Nevertheless, injection of 4T1 alone or in combination with MSCs^Gal-1−/−^ showed delayed tumor growth occurring between 21–32 days after initiation of the tumor (Fig. 3D). Examination of the survival of the animals in the different groups (Fig. S4) was carried out on limited number of mice due to sparing suffering of animals. Since one of five mice injected with 4T1 and wtMSC deceased as early as 38 days following tumor initiation, one mouse of each group was sacrificed before tumor-caused death for further analysis at the same time. Evaluation of the remaining animals' survival showed good correlation with the results of tumor incidence since all mice injected with 4T1 and wtMSC died within 45 days while those obtaining 4T1 or 4T1 and MSCs^Gal-1−^/died between 45 and 85 days after tumor initiation. Moreover 1 and 2 animals survived over 110 days in the groups injected with 4T1 and MSCs^Gal-1−/−^ and 4T1 alone, respectively.

To see whether MSC-derived Gal-1 enhanced tumor growth by contributing to establishment of tumor immuno-privilege similarly to tumor-cell derived Gal-1 did [18], [19], B16F10 melanoma cells were co-injected with wtMSCs or MSC^Gal-1−/−^ into X-SCID immuno-compromised mice. Gal-1 deficient MSCs failed to support tumor growth while wtMSCs effectively enhanced tumor size compared to tumor cells injected without MSCs (Fig. S5). These results indicated that MSC-derived Gal-1 did not act via the immune response when supported tumor growth.

### MSC-induced elevation of microvessel density of primary tumors requires Gal-1 expression by MSCs

**Figure 4:**
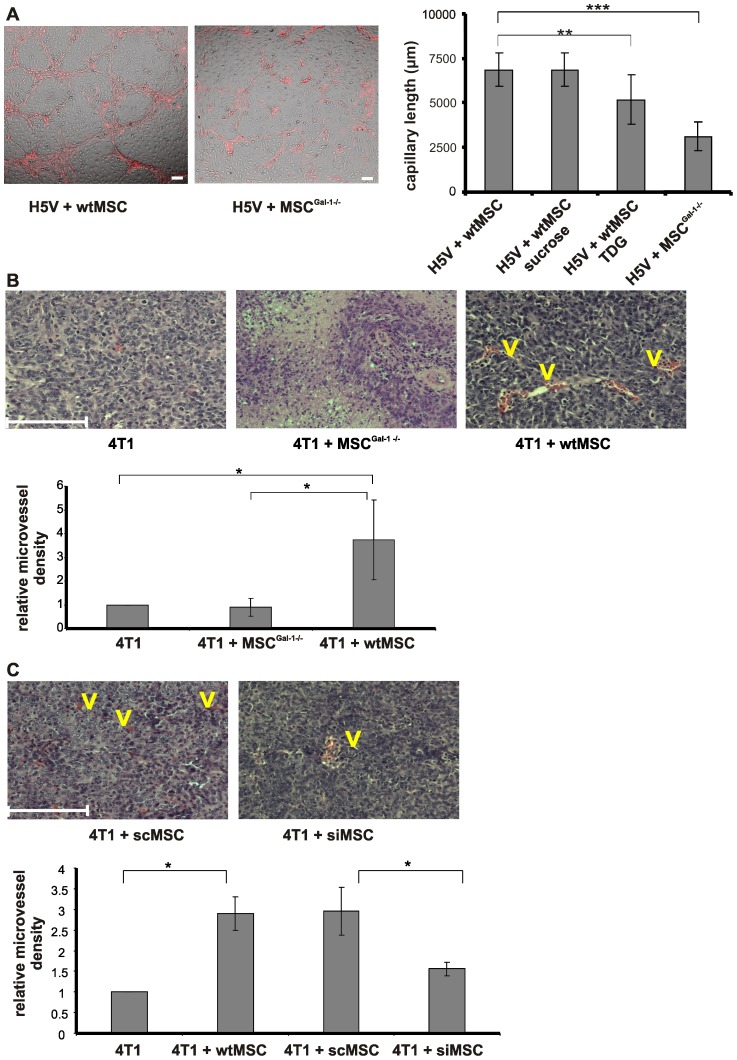
MSC-derived Gal-1 influences the capillary formation of H5V endothelioma *in vitro* and increases the microvessel density of 4T1 tumors *in vivo*. (A) *In vitro* capillary assay. CM-DiI labeled wtMSCs or MSC^Gal-1−/−^ (red) were incubated with mouse heart capillary endothelial cells (H5V) in a ratio of 1∶1 for 3 days (left) in the presence or absence of 30 mM sucrose or TDG. Tubes were quantified in parallel samples by measuring the length of capillary-like structures in five random areas of co-cultured cells (right). **p<0.001, ***p<0.0001. (B and C) *In vivo* analysis of microvessel density in tumors. Female Balb/C mice were challenged by 4T1 (10^3^ cells) alone (B, left) or in combination with 10^5^ MSC^Gal-1−/−^ (B, middle), wtMSC (B, right), scMSC (C, left) and siMSc (C, right). The presented microscopic pictures are representative of 3 experiments. Morphometric measurement of vascularized (V) areas (B and C lower panels) was performed on paraffin-embedded primary tumor tissue sections as described in *Materials and methods*. Scale bar is 100 µm. *p<0.05.

To find out whether Gal-1 was implicated in MSC-regulated tumor vascularization, an *in vitro* capillary assay was carried out. As shown on Fig. 4A, the absence of Gal-1 in MSCs resulted in diminished blood vessel-like structure formation when co-cultured with H5V murine endothelial cells as compared to the effect of wtMSCs. Neither MSCs nor H5V cells formed vessel-like structures alone (data not shown). Competition of Gal-1 effect with lactose analogue, thiodigalactoside (TDG), but not sucrose (control disaccharide) also resulted in significant reduction of capillary formation (Fig. 4A) confirming not only the role of Gal-1 in vessel-like structure formation, but also the function of Gal-1 localized extracellularly. Accordingly, breast carcinoma was vascularized similarly when tumor cells were applied alone or together with MSC^Gal-1−/−^. In contrast, wtMSCs dramatically increased the vascularization of the tumor (Fig. 4B). Interestingly, the vascularized area within the tumor showed significant decline when siMSCs were co-injected (Fig. 4C), supporting the reproducible but not significant decrease in the macroscopic parameters of the primary tumors (Fig. 3B). These results and the similar expression of other pro-angiogenic factors (VEGF, angiopoietin1 and ORP150, Fig. S3E and F) strongly indicated that Gal-1 in MSCs played an essential role in generating new capillary networks of the tumors. MSCs isolated from other source such as adipose tissue supported tumor vascularization on Gal-1 specific fashion (Fig. S6) similarly to bone marrow derived MSCs (Fig. 4).

### Gal-1 in MSCs is an important factor in promotion of tumor metastasis

**Figure 5:**
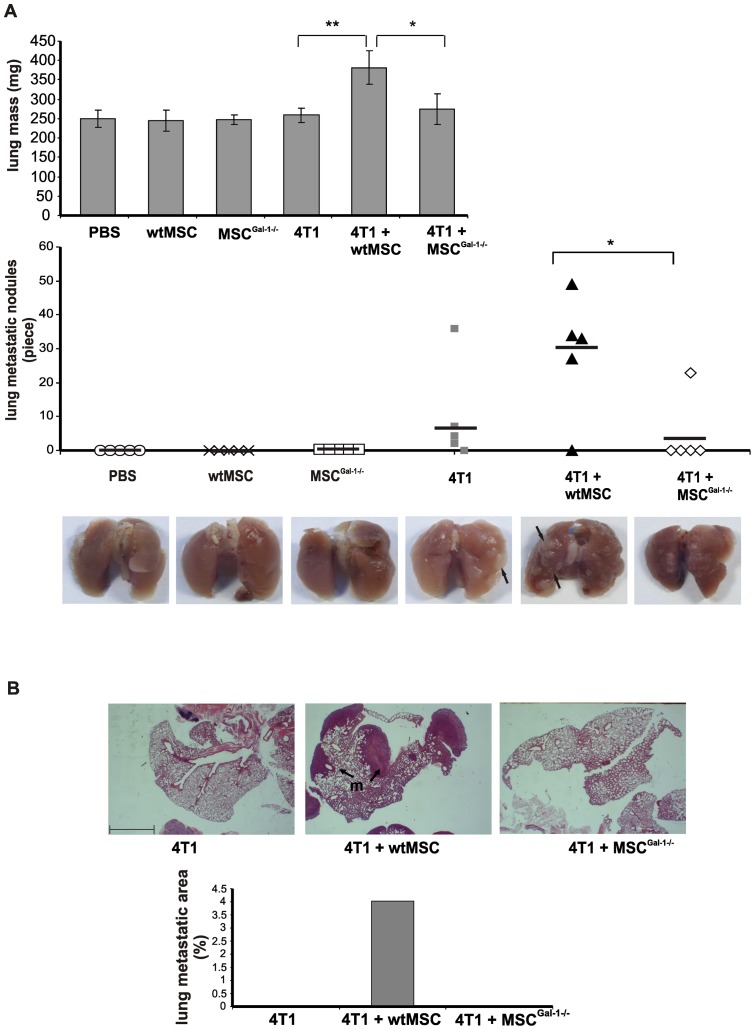
MSC-derived Gal-1 promotes tumor metastasis. Lungs were isolated from mice treated as described under Fig. 3. and fixed with formaldehyde. The lungs were weighed (A upper panel) and the metastatic nodules (marked with black arrows) were counted (A lower panel). * p<0.05, ** p<0.01, n = 5. Control mice were injected with either PBS, wtMSC or MSC^Gal-1−/−^. (B) Micro-metastases were evaluated on paraffin-embedded, haematoxylin-eosin-stained lung sections. Metastases (m) are delineated by black arrows. Percentage of lung metastatic area (lower graph) was determined as described in *Materials and methods*. Scale bar: 1 mm.

To determine the role of Gal-1 expression in MSCs regarding the frequency of lung metastasis, the lungs of differently treated animals were macroscopically surveyed (Fig. 5A) after sacrificing them. Average lung weights were around 250 mg in all experimental groups, except those from mice co-injected with 4T1 and wtMSCs which was significantly higher. Moreover, co-transplantation of wtMSCs resulted in a significant elevation of the number of lung metastatic nodules compared to that induced with MSC^Gal-1−/−^ or tumor cells alone.

Accordingly, histochemical analysis of the lung tissues showed that the ratio of the metastatic area *versus* the whole lung section isolated from wtMSCs co-injected animals were much higher than in lungs of animals transplanted with tumor cells alone or in combination with MSC^Gal-1−/−^ (Fig. 5B). These results implied that Gal-1 expression in MSCs efficiently contributed to promotion of metastasis.

### Tumorigenic effect of endogenous Gal-1 versus MSC-derived Gal-1

**Figure 6:**
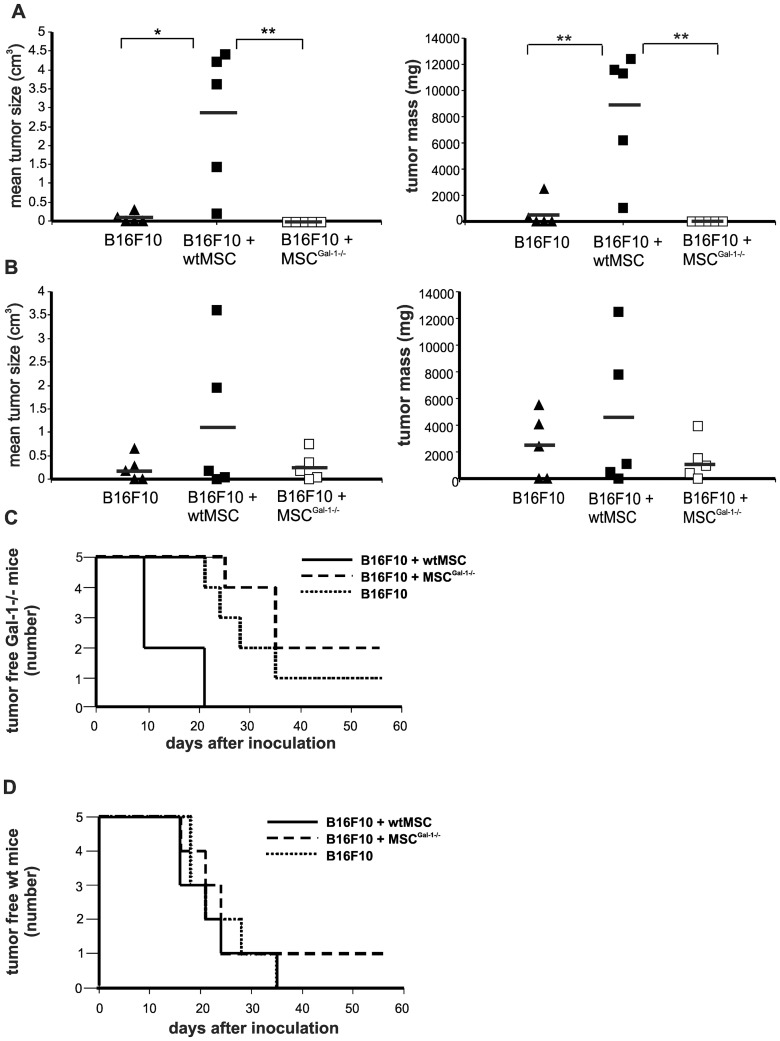
MSC-derived Gal-1 is sufficient to induce early tumor development in Gal-1 knockout mice. Five hundred B16F10 melanoma cells were subcutaneously injected alone or together with 10^5^ wtMSCs or MSC^Gal-1−/−^ into Gal-1 knockout (B6.Cg-*Lgals1* ^tm1Rob^/J) (A and C) or wild type C57BL/6 (B and D) mice. Tumor size (cm^3^) and tumor mass (mg) were measured on day 23 (left) and on day 24 (right), respectively (A and B). * p<0.05, ** p<0.01, n = 5. Kaplan-Meier analysis was carried out to evaluate the development of palpable tumors in Gal-1 knockout (C) and wt (D) mice.

To find out whether endogenous Gal-1 affected tumor growth we changed breast carcinoma to melanoma model to be able to use syngeneic tumor conditions. Wild type (wt) C57BL/6 or Gal-1 knockout (Gal-1^−/−^) B6.Cg-*Lgals1* ^tm1Rob^/J mice were treated with syngeneic B16F10 melanoma cells with or without wtMSCs or MSCs^Gal-1−/−^ (Fig. 6). Hardly detectable tumors were observed when Gal-1^−/−^ mice were injected with melanoma cells alone (Fig. 6A) compared to those in wt animals (Fig. 6B) on the 24^th^ day of injection. Co-transplantation of wtMSCs with melanoma cells accelerated tumor development in wild type mice although the enhancement was not statistically significant (Fig. 6B) in contrast to growth promoting effect of wtMSCs on the growth of breast carcinoma (Fig. 3A). The difference between the two tumor types could be attributed to the extremely high aggressiveness of melanoma. More importantly wtMSCs significantly and dramatically supported melanoma growth in Gal-1^−/−^ mice (Fig. 6A and C). In contrast, transplantation of tumor cells together with MSCs^Gal-1−/−^ did not promote tumor appearance until the 23^rd^ day in Gal-1 knockout mice (Fig. 6A). The presence of Gal-1 in MSCs seemed to be essential to support tumor development in knockout mice and Gal-1 expression in tumor cells was not sufficient to entirely by-pass the endogenous Gal-1 deficiency. Accordingly, tumor growth was urged in Gal-1^−/−^ mice injected with tumor together with wtMSCs, resulting in no tumor free animals within 21 days after transplantation. Those Gal-1 knockout animals which were injected with tumor cells alone or in the presence of Gal-1 deficient MSCs showed a delayed tumor growth as the first animals developed visible tumors on the 21^st^ and 25^th^ day and even after 60 days of observation one and two animals remained tumor free, respectively (Fig. 6C). Co-application of wtMSCs in wt mice hardly influenced the appearance of the tumor (Fig. 6D) indicating the aggressive growth of the melanoma.

## Discussion

Recently the stromal elements of solid tumors have obtained extensive attention since they serve as critical niche of the tumor tissue. Two elements of the tumor stroma, the tumor-associated fibroblasts (TAF) and myofibroblasts partially derive from bone marrow MSCs [3]. During tumor development, MSCs are mobilized into the circulation, migrate toward and engraft into the tumor microenvironment [36], [37]. In the tumors, MSCs favor tumorigenesis and tumor growth by differentiating into TAFs [12], [38], promoting neo-angiogenesis [11], [39] and establishing cancer stem cell niches with immunosuppressive effect [10], [14].

Opposite effects of exogenously administered MSCs on tumor progression have been recently demonstrated since MSCs supported or suppressed tumor growth [9]. Our findings are in accordance to those describing that MSCs promote tumor growth. Co-injection of MSCs with 4T1 breast carcinoma cells results in dramatic elevation of tumor size, weight, and tumor incidence while survival of the animals decrease. Moreover, transplantation of MSCs into pre-existing tumor bearing mice supports tumor growth on a fashion partially dependent of Gal-1 expression. In contrast, in melanoma model MSCs do not cause significant difference in tumor incidence compared to melanoma cells injected in the absence of MSCs demonstrating the high aggressiveness of the melanoma.

Tumor modulating effects of MSCs are attributed to numerous factors released into the tumor microenvironment [4]. One of the factors which MSCs produce and secrete is Gal-1 [25] however its function in tumor growth has not been revealed. Here we have identified Gal-1 as a novel, crucial factor in tumorigenic effect of MSCs. Tumor growth supporting function of Gal-1 knockout MSCs is significantly weaker compared to that of wild type MSCs.

MSCs are mobilized into the circulation upon hypoxia [40], a general feature of solid tumors, and attracted into the tumor by factors induced by hypoxia-induced factor1α (HIF1α), including VEGF, and SDF-1α [41]. Within the tumor, MSCs support tumor progression by enhancing neo-angiogenesis [42], contributing to the tumor immunoprivilege [10] and promoting metastasis [15]. Expression of Gal-1 is also regulated by hypoxia [43] and may serve as a mediator for all mentioned mechanisms including angiogenesis [22], [34]. We show that MSC-derived Gal-1 is implicated in formation of vessel-like structures *in vitro* since deficiency of Gal-1 expression in MSCs results in highly decreased tube formation in co-culture with H5V endothelial cells. *In vivo* data support these findings since wtMSCs increase microvessel density 4-fold, while MSC^Gal-1−/−^ does not affect it compared to the tumor triggered with breast cancer cells alone. This robust pro-angiogenic effect of MSC-derived Gal-1 becomes also obvious when siMSCs are applied together with breast carcinoma cells. Low amount of Gal-1 in the siMSCs is sufficient to partially sustain the overall tumor growth and a reduced tumor vascularization. It is of note, that tumor promoting effect of MSCs with reduced Gal-1 expression is between wtMSCs and Gal-1 deficient MSC^Gal-1−/−^. Our *in vitro* and *in vivo* results are supported by the data published by Burns et al [44]. These authors have shown that immortalized human MSCs recruit endothelial cells and decellularized matrix produced by MSCs promote vascularization on a Gal-1 dependent manner.

Mesenchymal stromal cells enhance tumor metastasis in breast carcinoma models implicating the role of pro-angiogenic CCL5 [15]. Gal-1 also increases the metastatic potential of tumor cells since Gal-1 expression in breast cancer-associated stroma cells shows significant correlation between tumor invasiveness and lymph node metastases [45]. As it is shown here, Gal-1 in MSCs also contributes to breast cancer metastasis. While wtMSCs significantly enhance metastasis, MSC^Gal-1−/−^ do not modulate these parameters compared to those induced by breast cancer cells alone. In an other aspect, Tsunoda et al. describe the effect of infiltrating T-cells to tumor stroma and eventually to neo-angiogenesis [46]. We transplanted X-SCID mice, in which the immunological aspect of the tumor growth regulation was excluded, with B16F10 melanoma and wt or Gal-1 KO MSCs. Absence of Gal-1 in MSCs resulted in the failure of tumor promotion by MSCs indicating that MSCs' tumor supporting effect did not directly depend on the regulation of tumor specific immune response or tumor infiltrating immune cells.

Galectin-1 expression in the tumor cells substitutes endogenous Gal-1 in some extent as demonstrated in Gal-1 knockout mice [23], [47]. Tumor cells producing Gal-1 allows more effective tumor angiogenesis compared to that of Gal-1 non-expressing tumor cells [22] in mice lacking endogenous Gal-1. Other authors argue that tumor cell-derived Gal-1 contributes to tumor growth and metastasis by regulating immunosuppression [47] even in Gal-1 knockout mice. In spite of the different emphasis, these papers and our results agree that Gal-1 expression within the tumor tissue strongly affect tumor growth and metastasis. Accordingly, we have found that Gal-1 expressing melanoma grows in Gal-1 knockout mice, although tumor development is impaired as compared to that of wild type animals. However, co-injection of wtMSCs but not MSC^Gal-1−/−^ with melanoma cells into Gal-1 knockout mice results in exaggerated tumor growth comparable to that in wild type animals treated with melanoma and wtMSC. These results show that simultaneous expression of Gal-1 in the tumor cells and exogenously transplanted MSCs completely restores the tumor-growth deficiency in Gal-1 knockout animals. In this scenario we hypothesize, that endogenous stromal elements deficient in Gal-1 expression may fail to support the vascularization, a prerequisite step of tumor establishment indicating a crucial role for endogenous stroma in tumor development. This theory is experimentally supported as *in vitro* formation of blood vessel-like structures is reduced in the absence of Gal-1 in MSCs. The importance of endogenous Gal-1 in tumor-promotion is substantiated with results obtained using Gal-1 knockout mice; while injection of tumor cells alone or in combination with MSC^Gal-1−/−^ results in highly delayed tumor appearance in Gal-1 knockout animals, co-transplantation of wtMSCs by-passes the absence of endogenous Gal-1. Our unpublished results (Krenács et al.) and other reported data [45], [48], [49] show that high Gal-1 expression in the stromal elements of breast and prostate carcinoma as well as gastric adenocarcinoma well correlates with the invasiveness and bad prognosis of the cancer disease. Accordingly, the results presented here support the theory that Gal-1 expression in MSCs is determining in assisting effective tumor growth since exogenously added wild type but not Gal-1 knockout MSC is able to overcome the delayed tumor growth in the absence of endogenous Gal-1 in the stromal elements of Gal-1 knockout mice.

Identification of individual factors playing role of MSC-mediated tumor promotion gains special interest as using MSCs in cell-mediated tumor therapy (reviewed by Dwyer [50]) is a novel and promising tool in cancer treatment. However, due to the frequently existing differences between mouse and human cell functions, the role of human MSC-derived Gal-1 in human tumor progression requires further validation.

## Supporting Information

Figure S1 **Characterization of MSCs.** wtMSC, MSC^Gal-1−/−^, scMSCs or siMSCs were labeled with R-Phycoerythrin conjugated monoclonal antibodies against CD44, CD73, CD90 and Sca-1(black lines) and analyzed with cytofluorimetry. Gray areas depict negative controls.
(TIF)

Figure S2 **Adipogenic and osteogenic differentiation of MSCs.** Wild type MSCs, MSC^Gal-1−/−^, scMSCs, siMSC were cultured in adipogenic (left panel) or osteogenic (right panel) medium. Lipid droplets and calcium deposits in the extracellular matrix were stained with Oil Red O and Alizarin Red S, respectively, and then analyzed with inverted light microscope. Scale bar: 50 µm.
(TIF)

Figure S3 **Expression of Gal-1 and pro-tumorogenic factors in MSCs and tumor cell lines.** Total cellular Gal-1 amounts in cell lysates prepared from wtMSCs, MSC^Gal-1−/−^, scMSCs and siMSCs (**A**) or from tumor cells, 4T1 breast carcinoma and B16F10 melanoma (**B**) were analyzed with Western blotting. Recombinant human Gal-1 (**B**) was used as a positive control. Gal-1 was developed with rabbit anti-Gal-1 followed by anti-rabbit Ig–HRP and ECL Plus. Rabbit anti-β-actin was used as loading control. (**C**) Gal-1 expression was compared in the used MSC and tumor cell lines analyzing the Gal-1 mRNA amount using QPCR. (**D**) Extracellular (wtMSCs: upper left, 4T1: lower left and B1610: lower right) and total (wtMSCs: upper right) Gal-1 was examined in wtMSCs with cytofluorimetry in unpermeabilized and permeabilized cells, respectively using goat anti-mouse Gal-1 and donkey anti-goat Ig-NL493. The cell were analyzed with cytofluorimetry. Expressions of COX2, TGF-β1, IL-10, VEGFA, angiopoietin1(**E**) and ORP150 and BEX2 (**F**) genes were analyzed with QPCR in wtMSCs and MSC^Gal-1−/−^.
(TIF)

Figure S4 **Wild type but not Gal-1^−/−^ MSCs reduce survival of mice with breast carcinoma.** Survival of mice challenged with 10^3^ 4T1 cells alone or in combination with 10^5^ wtMSCs or MSC^Gal-1−/−^ was evaluated using Kaplan-Meier analysis. Surviving of animals was surveyed up to 110 days. Number of animals in the experimental groups were: 4T1+wtMSC n = 5, 4T1+MSC^Gal-1−/−^ n = 4, 4T1 n = 4, wtMSC n = 4, MSC^Gal-1−/−^ n = 4, PBS n = 4.
(TIF)

Figure S5 **wtMSCs but not MSC^Gal-1−/−^ enhances the tumor growth in immune deficient X-SCID mice.** Five hundred B16F10 melanoma cells were injected subcutaneously alone or together with 10^5^ wtMSCs or MSC^Gal-1−/−^ into male X-SCID mice (n = 4 per group). Tumor size was monitored and calculated as described under Figure 3.
(TIF)

Figure S6 **Adipose tissue-derived MSCs increase the microvessel density of 4T1 tumors on a Gal-1 dependent fashion.** Female Balb/C mice were challenged by 4T1 (10^3^ cells) alone or in combination with 10^5^ A-MSC^Gal-1−/−^ or wtA-MSC. Morphometric measurement of vascularized areas was performed on paraffin-embedded primary tumor tissue sections as described in *Materials and methods*.
(TIF)

Table S1 **List of siRNA oligonucleotides.** Numbers in the name of oligonucleotides indicate the starting position of the 19 nucleotide long RNAi sequence in the human (hGalec) and murine galectin-1 (mGalec) mRNA and the appropriate scrambled controls.
(TIF)
