# Supplementary figures and images for: Identification of Galectin-1 as a Critical Factor in Function of Mouse Mesenchymal Stromal Cell-Mediated Tumor Promotion
Source: PLoS One. 2012 Jul 23;7(7):e41372. doi: 10.1371/journal.pone.0041372 (PMC3402504; doi:10.1371/journal.pone.0041372)

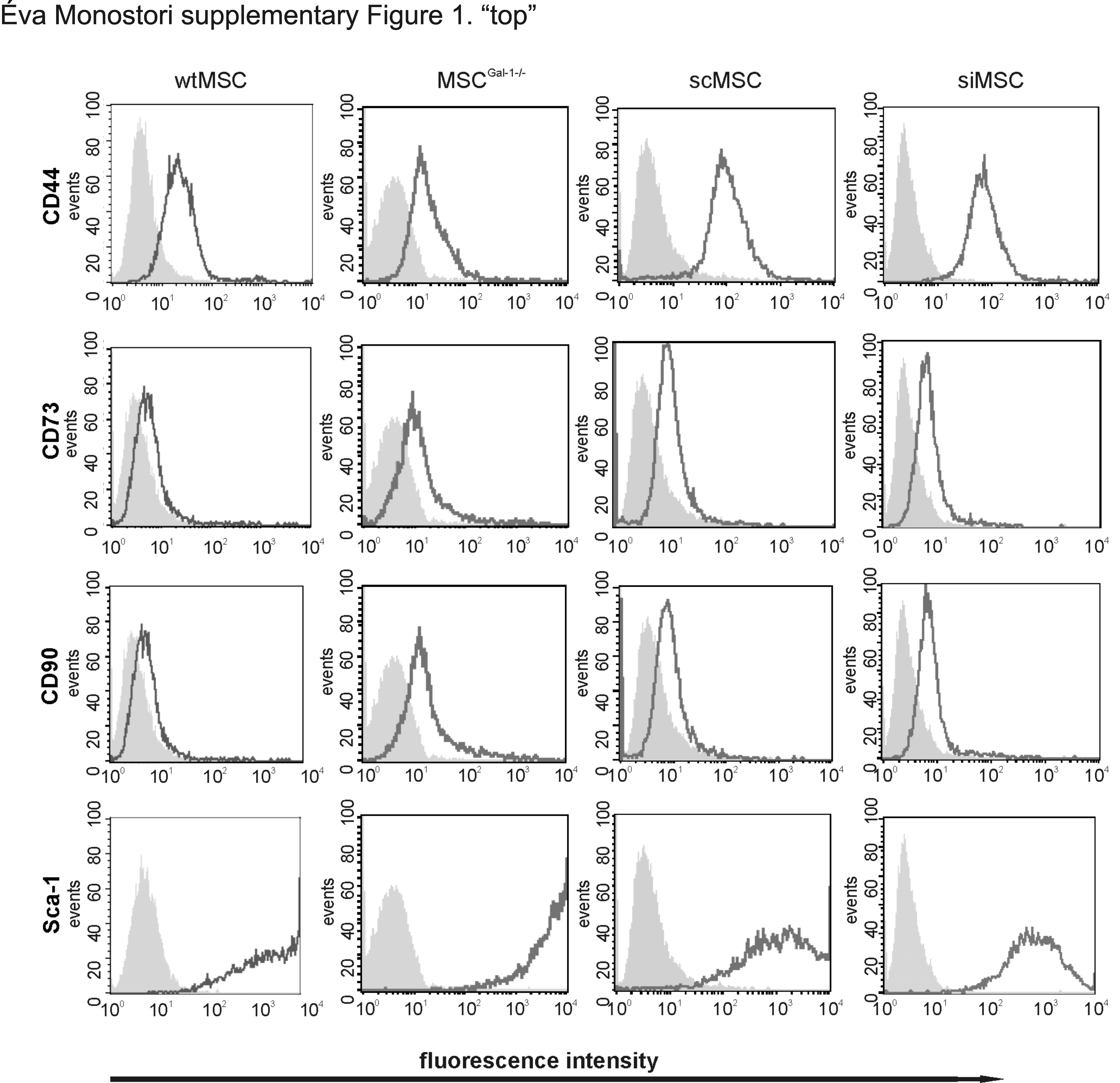

Supplement: Figure S1 — Characterization of MSCs. wtMSC, MSCGal-1−/−, scMSCs or siMSCs were labeled with R-Phycoerythrin conjugated monoclonal antibodies against CD44, CD73, CD90 and Sca-1(black lines) and analyzed with cytofluorimetry. Gray areas depict negative controls. (TIF) [file pone.0041372.s001.tif]

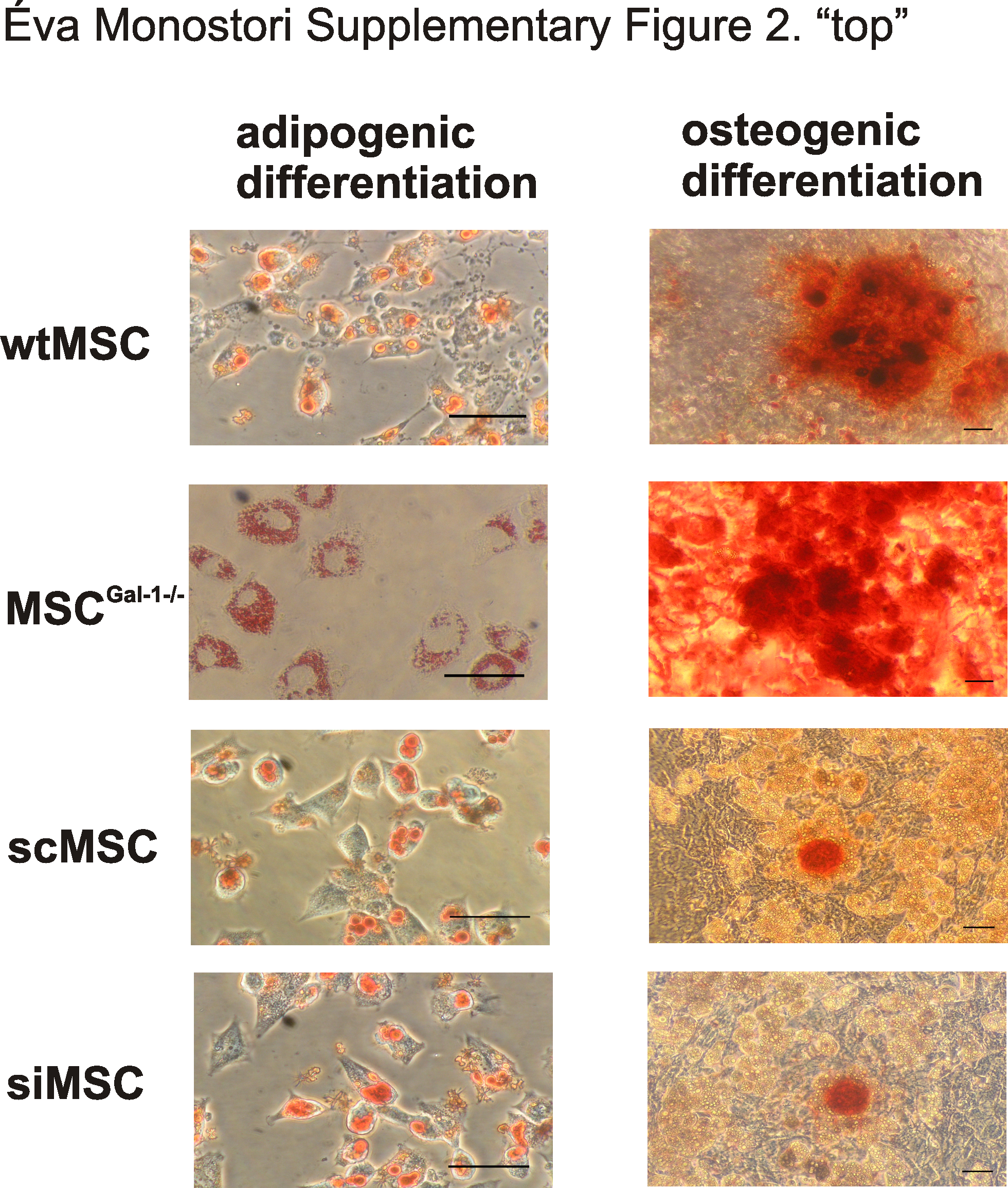

Supplement: Figure S2 — Adipogenic and osteogenic differentiation of MSCs. Wild type MSCs, MSCGal-1−/−, scMSCs, siMSC were cultured in adipogenic (left panel) or osteogenic (right panel) medium. Lipid droplets and calcium deposits in the extracellular matrix were stained with Oil Red O and Alizarin Red S, respectively, and then analyzed with inverted light microscope. Scale bar: 50 µm. (TIF) [file pone.0041372.s002.tif]

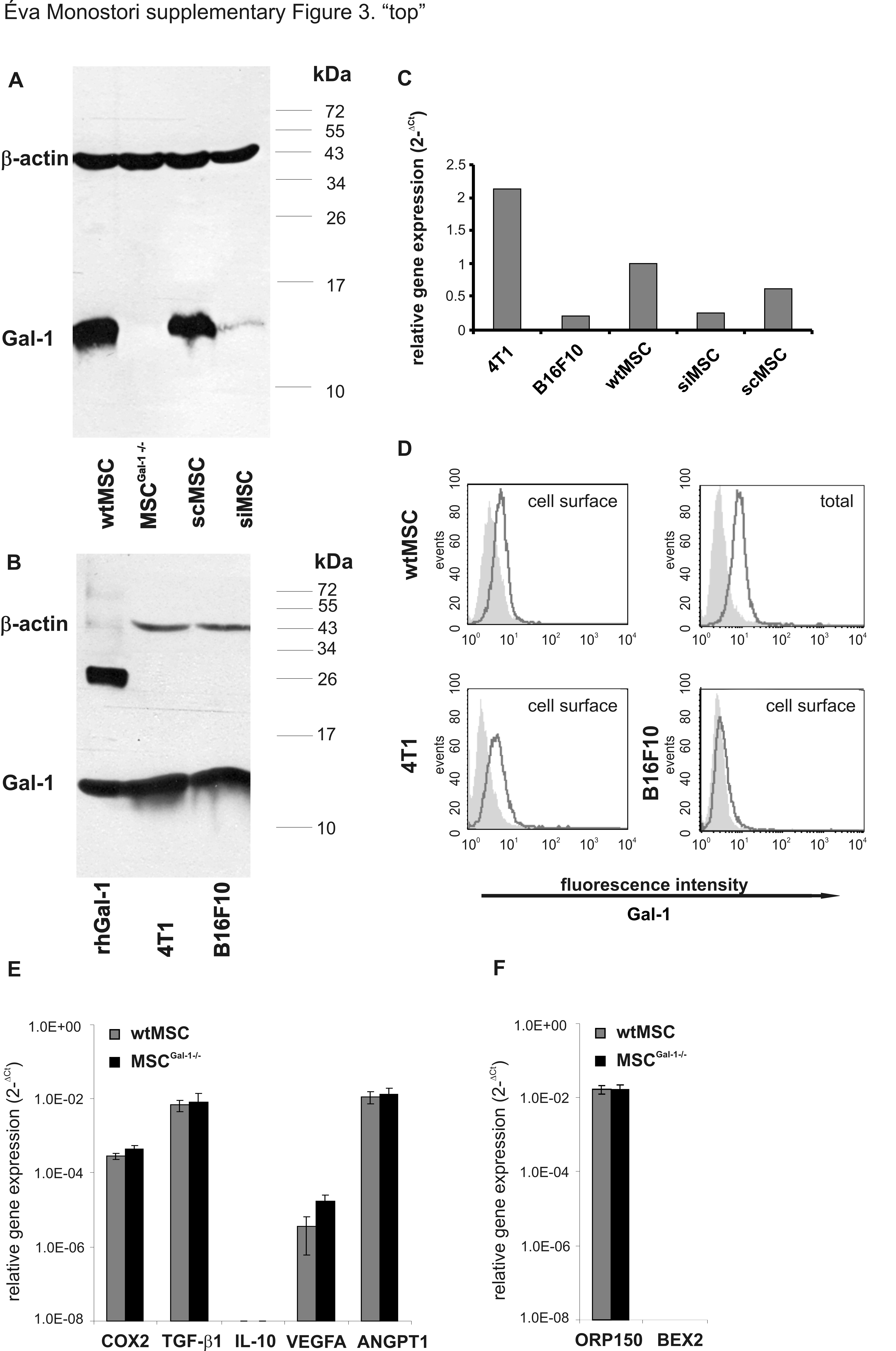

Supplement: Figure S3 — Expression of Gal-1 and pro-tumorogenic factors in MSCs and tumor cell lines. Total cellular Gal-1 amounts in cell lysates prepared from wtMSCs, MSCGal-1−/−, scMSCs and siMSCs (A) or from tumor cells, 4T1 breast carcinoma and B16F10 melanoma (B) were analyzed with Western blotting. Recombinant human Gal-1 (B) was used as a positive control. Gal-1 was developed with rabbit anti-Gal-1 followed by anti-rabbit Ig–HRP and ECL Plus. Rabbit anti-β-actin was used as loading control. (C) Gal-1 expression was compared in the used MSC and tumor cell lines analyzing the Gal-1 mRNA amount using QPCR. (D) Extracellular (wtMSCs: upper left, 4T1: lower left and B1610: lower right) and total (wtMSCs: upper right) Gal-1 was examined in wtMSCs with cytofluorimetry in unpermeabilized and permeabilized cells, respectively using goat anti-mouse Gal-1 and donkey anti-goat Ig-NL493. The cell were analyzed with cytofluorimetry. Expressions of COX2, TGF-β1, IL-10, VEGFA, angiopoietin1(E) and ORP150 and BEX2 (F) genes were analyzed with QPCR in wtMSCs and MSCGal-1−/−. (TIF) [file pone.0041372.s003.tif]

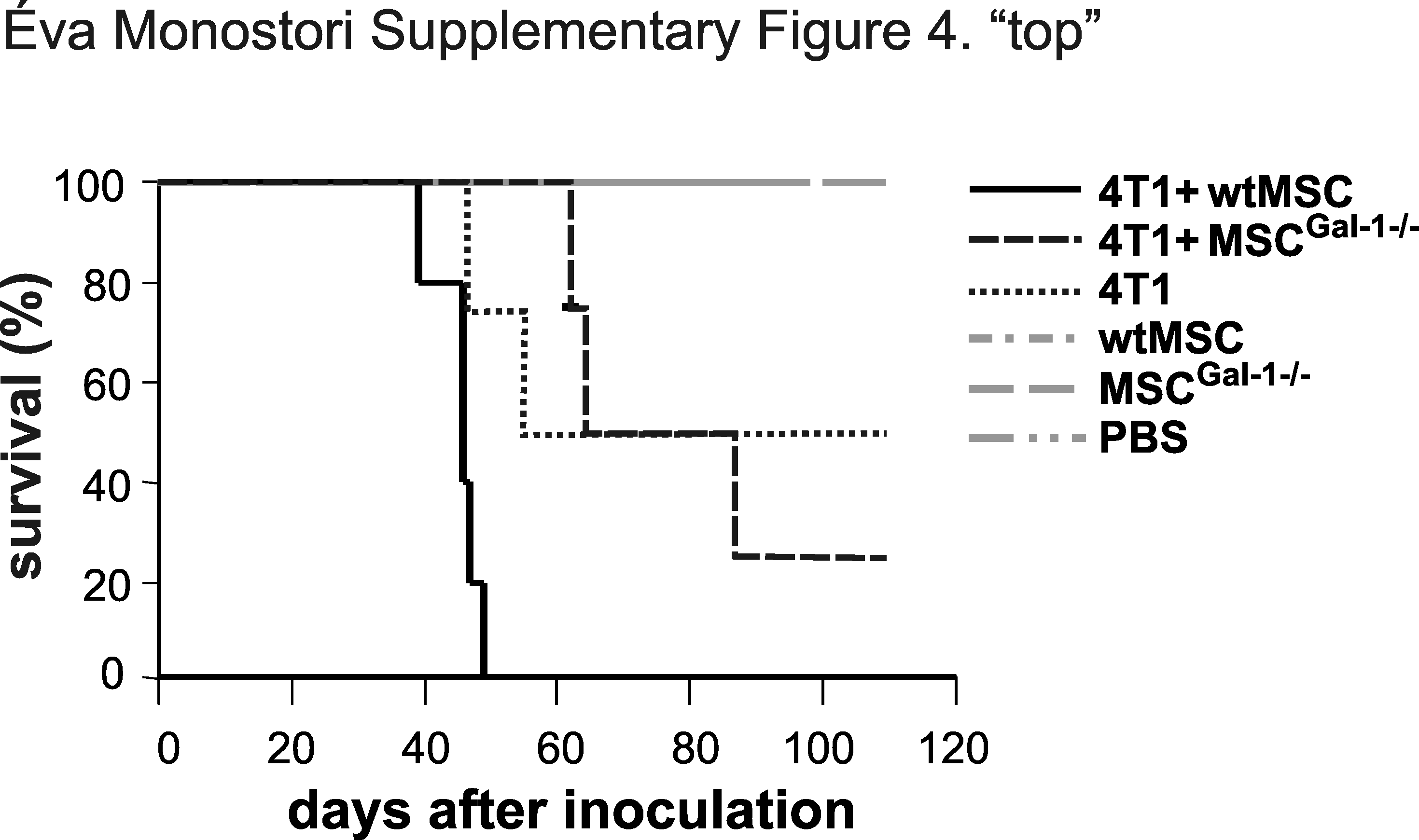

Supplement: Figure S4 — Wild type but not Gal-1−/− MSCs reduce survival of mice with breast carcinoma. Survival of mice challenged with 103 4T1 cells alone or in combination with 105 wtMSCs or MSCGal-1−/− was evaluated using Kaplan-Meier analysis. Surviving of animals was surveyed up to 110 days. Number of animals in the experimental groups were: 4T1+wtMSC n = 5, 4T1+MSCGal-1−/− n = 4, 4T1 n = 4, wtMSC n = 4, MSCGal-1−/− n = 4, PBS n = 4. (TIF) [file pone.0041372.s004.tif]

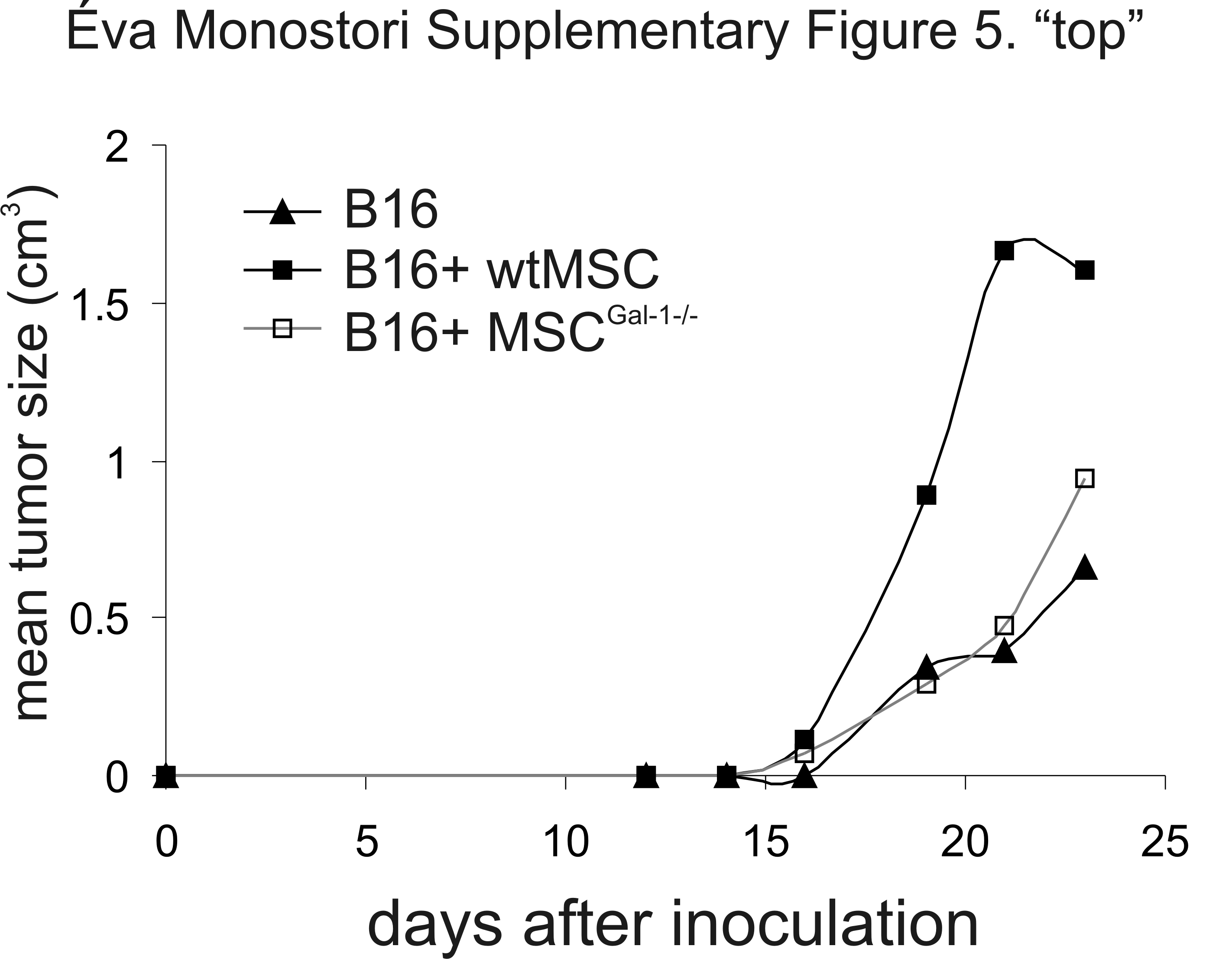

Supplement: Figure S5 — wtMSCs but not MSCGal-1−/− enhances the tumor growth in immune deficient X-SCID mice. Five hundred B16F10 melanoma cells were injected subcutaneously alone or together with 105 wtMSCs or MSCGal-1−/− into male X-SCID mice (n = 4 per group). Tumor size was monitored and calculated as described under Figure 3. (TIF) [file pone.0041372.s005.tif]

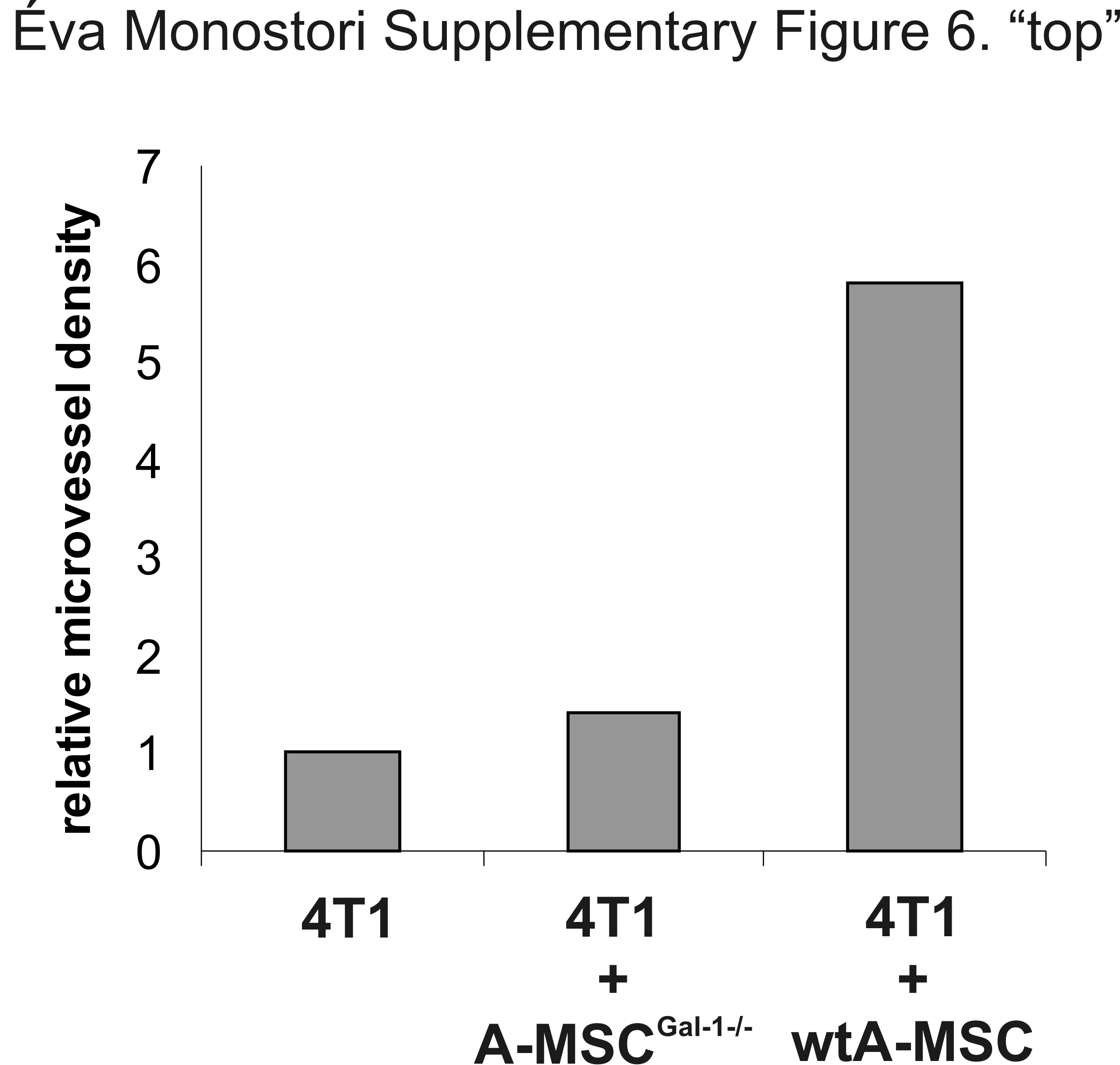

Supplement: Figure S6 — Adipose tissue-derived MSCs increase the microvessel density of 4T1 tumors on a Gal-1 dependent fashion. Female Balb/C mice were challenged by 4T1 (103 cells) alone or in combination with 105 A-MSCGal-1−/− or wtA-MSC. Morphometric measurement of vascularized areas was performed on paraffin-embedded primary tumor tissue sections as described in Materials and methods. (TIF) [file pone.0041372.s006.tif]

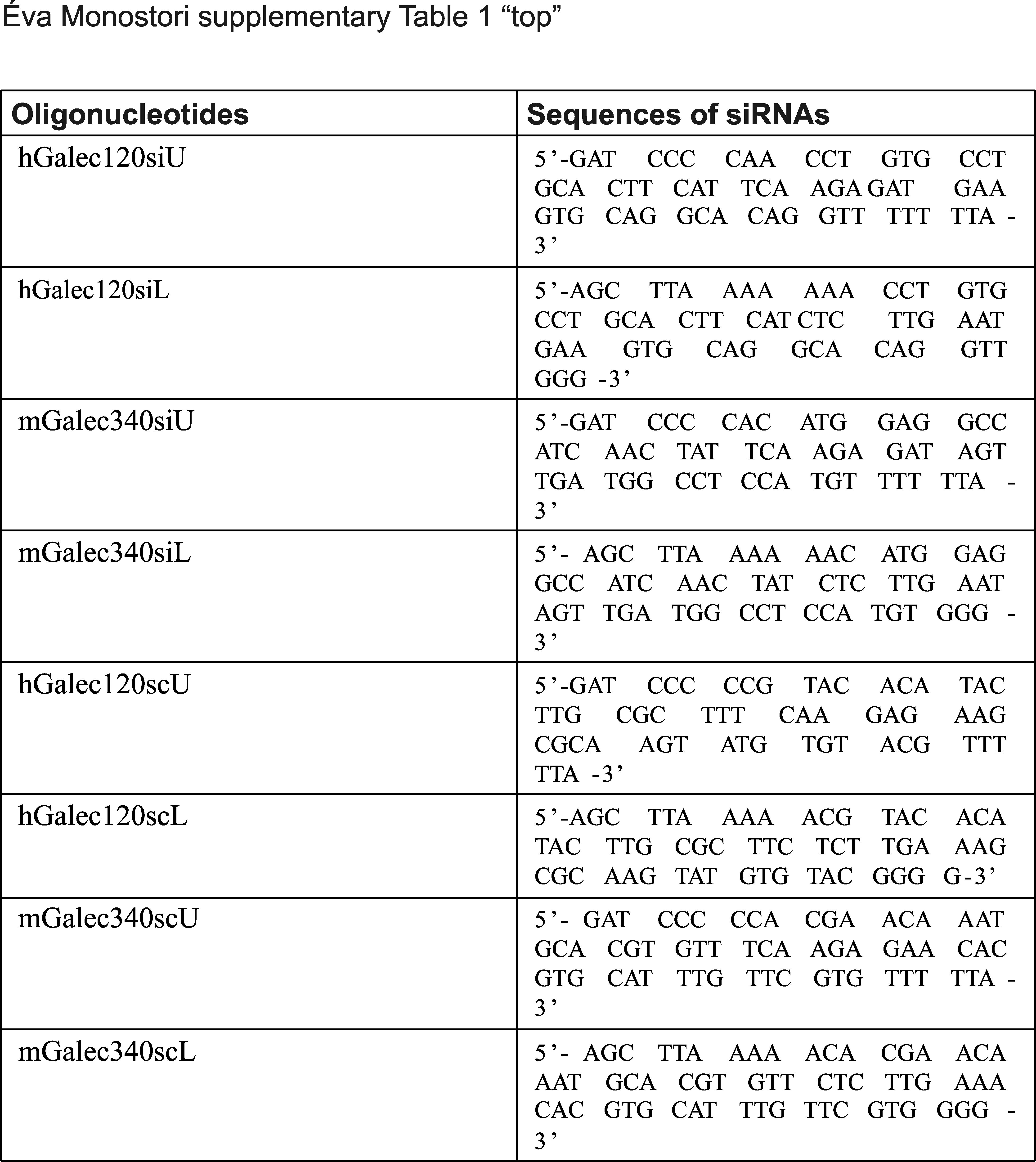

Supplement: Table S1 — List of siRNA oligonucleotides. Numbers in the name of oligonucleotides indicate the starting position of the 19 nucleotide long RNAi sequence in the human (hGalec) and murine galectin-1 (mGalec) mRNA and the appropriate scrambled controls. (TIF) [file pone.0041372.s007.tif]
